# [Retracted] Farnesoid X receptor inhibits proliferation of human colorectal cancer cells via the miR‑135A1/CCNG2 signaling pathway

**DOI:** 10.3892/or.2025.8896

**Published:** 2025-04-14

**Authors:** Pengfei Qiao, Shenglong Li, Haogang Zhang, Lei Yao, Fujing Wang

Oncol Rep 40: 2067–2078, 2018; DOI: 10.3892/or.2018.6636

Following the publication of the above article, a concerned reader drew to the Editor's attention that there were potential anomalies associated with the colony formation assay data shown in Fig. 5H on p. 2076; essentially, concerning the SW620 data (bottom row), and comparing the first and third images from the left, groupings of cells appeared to be markedly similar in terms of their positioning. Patternings of subgroups of cells within the images were very similar, with cells occupying matching positions in the images, to the extent that it was difficult to conceive of the similarities as being coincidental. This phenomenon was also noted to apply to the equivalent images (bottom row, first and third images from the left) for the SW620 data in Fig. 4D.

After having conducted an internal investigation of the data in this paper, the Editor of *Oncology Reports* has judged that the potentially anomalous presentation of the strikingly similar groupings of cells in Figs. 4D and 5H was too extensive that these features could have been easily attributed to pure coincidence. Therefore, the Editor has decided that this article should be retracted from the publication on the grounds of an overall lack of confidence in the data. The authors were asked for an explanation to account for these concerns, but the Editorial Office did not receive a satisfactory reply. The Editor sincerely apologizes to the readership for any incovenience caused, and we thank the reader for bringing this matter to our attention.

